# Elevated Serum Vascular Cell Adhesion Molecule-1 Is Associated with Septic Encephalopathy in Adult Community-Onset Severe Sepsis Patients

**DOI:** 10.1155/2014/598762

**Published:** 2014-05-06

**Authors:** Chih-Min Su, Hsien-Hung Cheng, Tsung-Cheng Tsai, Sheng-Yuan Hsiao, Nai-Wen Tsai, Wen-Neng Chang, Wei-Che Lin, Ben-Chung Cheng, Yu-Jih Su, Ya-Ting Chang, Yi-Fang Chiang, Chia-Te Kung, Cheng-Hsien Lu

**Affiliations:** ^1^Department of Biological Science, National Sun Yat-Sen University, Kaohsiung 80424, Taiwan; ^2^Department of Emergency Medicine, Kaohsiung Chang Gung Memorial Hospital, Chang Gung University College of Medicine, No. 123, Ta Pei Road, Niao Sung Hsiang, Kaohsiung 833, Taiwan; ^3^Department of Neurology, Kaohsiung Chang Gung Memorial Hospital, Chang Gung University College of Medicine, No. 123 Ta Pei Road, Niao Sung Hsiang, Kaohsiung 833, Taiwan; ^4^Department of Radiology, Kaohsiung Chang Gung Memorial Hospital, Chang Gung University College of Medicine, No. 123, Ta Pei Road, Niao Sung Hsiang, Kaohsiung 833, Taiwan; ^5^Department of Internal Medicine, Kaohsiung Chang Gung Memorial Hospital, Chang Gung University College of Medicine, No. 123, Ta Pei Road, Niao Sung Hsiang, Kaohsiung 833, Taiwan

## Abstract

*Background and Aim*. Septic encephalopathy (SE) is a common complication of severe sepsis. Increased concentrations of circulating soluble adhesion molecules are reported in septic patients. This study aimed to determine whether serum adhesion molecules are associated with SE. *Methods*. Seventy nontraumatic, nonsurgical adult patients with severe sepsis admitted through ER were evaluated. Serum adhesion molecules were assessed for their relationship with SE, and compared with other clinical predictors and biomarkers. *Results*. Twenty-three (32.8%) patients had SE. SE group had higher in-hospital mortality (40% versus 11%, *P* = 0.009) and their sVCAM-1, sICAM-1, and lactate levels on admission were also higher than non-SE group. By stepwise logistic regression model, sVCAM-1, age, and maximum 24-hours SOFA score were independently associated with septic encephalopathy. The AUC analysis of ROC curve of different biomarkers showed that sVCAM-1 is better to predict SE. The sVCAM-1 levels in the SE group were significantly higher than those of the non-SE group at three time periods (Days 1, 4, and 7). *Conclusions*. Septic encephalopathy implies higher mortality in nontraumatic, nonsurgical patients with severe sepsis. VCAM-1 level on presentation is a more powerful predictor of SE in these patients than lactate concentration and other adhesion molecules on admission.

## 1. Introduction

Septic encephalopathy (SE) is a common complication of severe sepsis and septic shock. An estimated 9–71% of patients with sepsis exhibit symptoms of encephalopathy [1–3], including consciousness disturbance, impaired cognitive function, personality changes, and lack of concentration or somnolence [4, 5]. Several mechanisms have been proposed, including oxidative stress [6], increased cytokine and proinflammatory factor [7], mitochondrial dysfunction, apoptosis [8], decreased cerebral blood flow [9], endothelium activation [10], and blood-brain barrier breakdown [11], as well as any combination of the abovementioned etiologies. By itself, SE is pivotal in determining sepsis mortality [12] and makes clinical evaluation more complex since patients cannot express themselves well. Although SE has been described as a reversible syndrome, studies indicate long-lasting cognitive and depressive disturbances in patients after the sepsis resolves [13]. Early accurate detection of septic encephalopathy not only makes clinical physician more alert about the sepsis status and aggressive treatment but also decreases unnecessary examination and movement of patient.

To date, useful biomarkers in predicting SE are still limited. Several studies used S-100B protein, a marker of astrocytes activation and injury, as a marker for brain injury in SE [14, 15]. However, its results were not promising. Five studies demonstrated that elevated S-100B protein correlated with the development of SE, but two other studies found no correlation and all their sample sizes were small [16, 17]. On the other hand, studies using animal models showed that some proinflammatory cytokines or cell adhesion molecules could be potential biomarkers that induce SE, but their effects on human beings are still not demonstrated [18–20].

In human studies, increased concentrations of circulating soluble adhesion molecules have been reported in patients with systemic inflammatory response syndrome, septic shock, and cardiovascular diseases [21, 22]. Increased concentrations of adhesion molecules have also been associated with multiple organ dysfunction, disease severity, or death [23]. Since sVCAM-1 and sICAM-1 have been involved in leukocyte-endothelium cell crosstalk at the blood-brain barrier [24], we wanted to know their relationship with SE. This prospective study aimed to determine the roles of serum adhesion molecules and conventional biomarkers in predicting SE among adult severe sepsis patients.

## 2. Patients and Methods

### 2.1. Study Population

This is a secondary analysis of prospective collected data on the time course of levels of adhesion molecules in severe sepsis and septic shock patients and the association of these biomarkers with SE. Over an 18-month period (January 2011 to June 2012), patients aged ≥20 years who were admitted through emergency room (ER) of Kaohsiung Chang Gung Memorial Hospital (CGMH), a 2482-bed acute-care teaching hospital in southern Taiwan providing both primary and tertiary referral care, were screened every weekday for severe sepsis and septic shock according to specific criteria and were enrolled in the study within 24 hours after identification. The hospital's Institutional Review Committee on Human Research approved the study, and all of the patients provided informed consent.

Severe sepsis on ER admission was defined according to the American College of Chest Physicians/Society of Critical Care Medicine criteria, which included the following: (a) suspicion or confirmed infection; (b) two or more manifestations of systemic inflammatory response syndrome; and (c) at least one sepsis-induced acute organ dysfunction or signs of hypoperfusion. All of the patients who met these three criteria were eligible to enroll in the study. Septic shock was defined as severe sepsis associated with hypotension not controlled by vascular expansion but requiring vasopressive agents to maintain SBP > 90 mmHg [25].

Patients were excluded if they had one of the following: (1) traumatic etiology; (2) previous surgical treatment; (3) underlying hematologic diseases or those under chemotherapy; (4) pregnancy; (5) central nervous systems disorders with various levels of conscious disturbance before arriving at the ER; and (6) history of exposure to drug, toxic substances, alcohol, industrial agents, heavy metals, or any substance known to cause consciousness change. We excluded these patients to eliminate the influence of inflammation reaction other than sepsis on the biomarkers and reduced the potential factors that would affect the diagnosis of SE.

### 2.2. Clinical Assessment and Treatment

The patient information collected was demographic data, Acute Physiology and Chronic Health Evaluation (APACHE) II score, Sequential Organ Failure Assessment (SOFA) score, and Charlson Comorbidity Index (CCI) score, which were calculated during the first 24 hours of admission to assess the severity of organ dysfunction. Basic laboratory tests, lactate concentration, B-type natriuretic peptide, and inflammatory markers, including plasma C-reactive protein (CRP) and procalcitonin, were taken on ER admission. The course of various organ dysfunctions and supportive treatments like vasoactive drugs, ventilator, and steroid therapies were also recorded.

### 2.3. Definition

Comorbidity was defined as preexisting disorder before severe sepsis event. Stroke history in this study only referred to the patients who still could maintain clear conscious level after ischemic or hemorrhagic stroke event. Charlson comorbidity scoring system was used to assess the severity of comorbidity [26]. The source of infection was classified as one of the following: lower respiratory tract, urinary tract, skin and musculoskeletal soft tissue, central nervous system, or intra-abdomen infection which included liver abscess, spontaneous bacterial peritonitis, biliary tract, peritonitis, and primary bacteraemia with unknown focus. For the grading of disease severity, we use both 24 h APACHE II and 24 h SOFA score which were calculated according to their laboratory data and clinical parameters [27, 28]. And mortality in this study means in-hospital mortality. Ventilator treatment within 24 hours means that the patient needed ventilator support to help him or her maintain adequate oxygenation and the event happened within 24 hours of ER admission.

### 2.4. Septic Encephalopathy

The patient's conscious level was recorded by the Glasgow Coma Scale (GCS) and mental status at least twice daily. Symptoms of SE included somnolence, stupor, coma, confusion, disorientation, agitation, irritability, and decreased level of GCS. Encephalopathy was confirmed if the patient had two or more of the aforementioned symptoms for more than 72 hours, regained consciousness after treatment, or deteriorated and died. Continuous sedative medication was never used, even in mechanically ventilated patients, although short-duration sedatives were used if patients did not cooperate for the treatment and examination. Patients with obvious etiologies of consciousness change other than SE during treatment were excluded. These include severe hypoglycemia event, intracranial hemorrhage, status epilepsy, acute ischemic stroke, hyponatremia, and cardiopulmonary resuscitation related hypoxic encephalopathy.

### 2.5. Assessment of Infectious Biomarkers

All tests were conducted by the quality-controlled central laboratory of CGMH. Concentrations of CRP were determined by enzyme immunoassay (EMIT; Merck Diagnostica; Zurich, Switzerland), while PCT was measured using enzyme-linked fluorescent assay (VIDAS; BioMerieux; Ponte a Ema, Italy). Serum lactate levels were measured using a serum-based assay catalyzed by lactate oxidase (UniCel Integrated System; Beckman Coulter INS; Boulevard, Brea, CA).

### 2.6. Blood Sampling and Assessment of Serum Adhesion Molecules

Blood samples of serum adhesion molecules were collected on the first day of enrollment (Day 1). Additional samples were obtained on Days 4 and 7. Blood samples were collected by venipuncture into Vacutainer SST tubes. Blood was allowed to clot in room temperature for a minimum of 30 minutes, and the clot was removed by immediate centrifugation at 3,000 rpm for 10 min at 4°C. All serum samples were collected after centrifugation, isolated, and stored at −80°C in multiple aliquots. Serum sICAM-1, sVCAM-1, sE-selectin, sL-selectin, and sP-selectin levels were determined by commercially available ELISA (R&D Systems, Minneapolis, MN, USA). In the assay, standards, controls, and unknown samples were incubated in microtitration wells that were coated with marked antibodies (i.e., anti-ICAM-1, VCAM-1, P-selectin, E-selectin, and L-selectin). After incubation and washing, the wells were treated with another anti-Ag detection antibody labeled with enzyme horseradish peroxidase (HRP).

After second incubation and washing, the wells were incubated with the substrate tetramethylbenzidine (TMB). An acidic stopping solution was then added, and the enzymatic turnover rate of the substrate was determined by dual wavelength absorbance measured at 450 and 620 nm. Absorbance was directly proportional to the concentration of antigens present. A set of antigen standards was used to plot a standard curve of absorbance versus antigen concentration, from which antigen concentrations in the unknowns were calculated.

Samples for serum adhesion molecules were collected and measured immediately. It takes 5 hours to measure serum levels. Other serum samples were collected after centrifugation, isolated, and stored at −80°C in multiple aliquots in accordance with our study design [29].

### 2.7. Statistical Analysis

Data were presented as mean ± SD or *n* (%) accordingly. Comparisons between septic encephalopathy and no encephalopathy groups were made by Mann-Whitney *U* test, while proportions among groups were compared by using *χ* ^2^ test or Fisher's exact test. Repeated measures of ANOVA were used to compare serum adhesion molecules at three different time points after severe sepsis. Analysis of covariance (ANCOVA) was used to compare groups after controlling for potential confounding variables. Spearman correlation analysis was used to test the correlation of serum adhesion molecule levels and traditional infection makers with the severity of sepsis, including maximum 24 h SOFA score and maximum 24 h APACHE II score.

Stepwise logistic regression was used to evaluate the relationship between significant variables and septic encephalopathy, with adjustments for other potential confounding factors. Only variables strongly associated with septic encephalopathy (*P* < 0.05) were included in the final model. Receiver operating characteristic (ROC) curves were generated to determine a cut-off level for significant variables for SE. Areas under the ROC curves (AUCs) were calculated for each parameter and compared. All statistical analyses were conducted using the SAS software package, version 9.1 (2002, SAS Statistical Institute, Cary, NC).

## 3. Results

### 3.1. Baseline Characteristics of the Study Patients

Seventy-five adult severe sepsis and septic shock patients were enrolled, but five patients were excluded after finding the etiology of consciousness change. Two of them had severe hypoglycemia, and the other 3 patients had severe hyponatremia, basilar artery occlusion as revealed by brain magnetic resonance imaging, and status epilepsy by electroencephalography, respectively. In the 70 patients, there were 22 females and 48 males, with an average age of 64.3 years. During admission, 23 patients experienced SE and 47 patients did not. Among the 23 SE events, 14 (61%) occurred at the time of enrollment, 6 (26%) were within 24 h after enrollment, and 3 (13%) were after 24 h but within 3 days of enrollment. The 14 patients who received brain computed tomography (CT) examination all had nonspecific findings. Four patients died after SE, but another five patients had returned to clear consciousness before occurrence of death.

Baseline characteristics, including comorbidities, clinical presentations, hospital mortality, and disease severity index including shock within 24 h, mechanical ventilation treatment within 24 h, and maximum 24 h APACHE II and 24 h SOFA scores between the SE and non-SE groups, were listed in Table 1. In this study, the patient with a stroke history was more likely to have SE (odds ratio (OR) 5.6, *P* = 0.03). In the disease severity index, the SE group had higher APACHE II and SOFA scores (21.3 ± 5.5 versus17.5 ± 5.7, *P* = 0.01; 8.2 ± 2.4 versus 5.4 ± 3.1, *P* < 0.001, resp.), which meant more organ dysfunction. In-hospital mortality and ventilator treatment within 24 hours were also both higher in SE patients (40% versus 11%, *P* = 0.009; 57% versus 24%, *P* = 0.008, resp.).

The sources of infection and laboratory data of the two groups were listed in Tables 2 and 3, respectively. There was no significant difference between infection source and culture result. Serum lactate (50.5 ± 37.6 versus 32.6 ± 20.3, *P* = 0.05), sICAM-1 (1028.2 ± 525.2 versus 764.8 ± 504.9, *P* = 0.03), and sVCAM-1_Day1_ (3048.1 ± 1261.1 versus 1969.0 ± 1129.5, *P* = 0.001) were the only three markers with significant difference between the SE and non-SE groups.

### 3.2. Effect of Infection Markers and Serum Adhesion Molecules on Sepsis Severity

Based on the statistical results (Spearman correlation coefficient, *P* value), sICAM-1_Day1_ level (*ρ* = 0.36, *P* = 0.003), sVCAM-1_Day1_ level (*ρ* = 0.404, *P* = 0.001), sE-selectin_Day1_ (*ρ* = 0.364, *P* = 0.002), procalcitonin (*ρ* = 0.347, *P* = 0.004), and lactate (*ρ* = 0.379, *P* = 0.001) had correlation with maximum 24 h SOFA score. sE-selectin_Day1_ (*ρ* = 0.284, *P* = 0.02), sVCAM-1_Day1_ (*ρ* = 0.287, *P* = 0.018), and lactate (*ρ* = 0.441, *P* < 0.001) had correlation with maximum 24 h APACHE II score. Traditional infection markers, CRP, were neither related to SOFA nor to APACHE II score.

### 3.3. Prediction of Septic Encephalopathy

Serum sVCAM-1_Day1_ level, sICAM-1_Day1_ level, serum lactate level, 24 h SOFA score, 24 h APACHE II score, stroke history, and ventilator treatment within 24 hours were significantly higher in SE patients and could be used as clinical predictors. However, after using both forward and backward stepwise logistic regression model with all the predictors plus age and sex, only sVCAM-1_Day1_ level (*P* = 0.009, 0.02), age (*P* = 0.002, 0.011), and SOFA score (*P* = 0.007, 0.002) were independently associated with SE.

**Figure 1:**
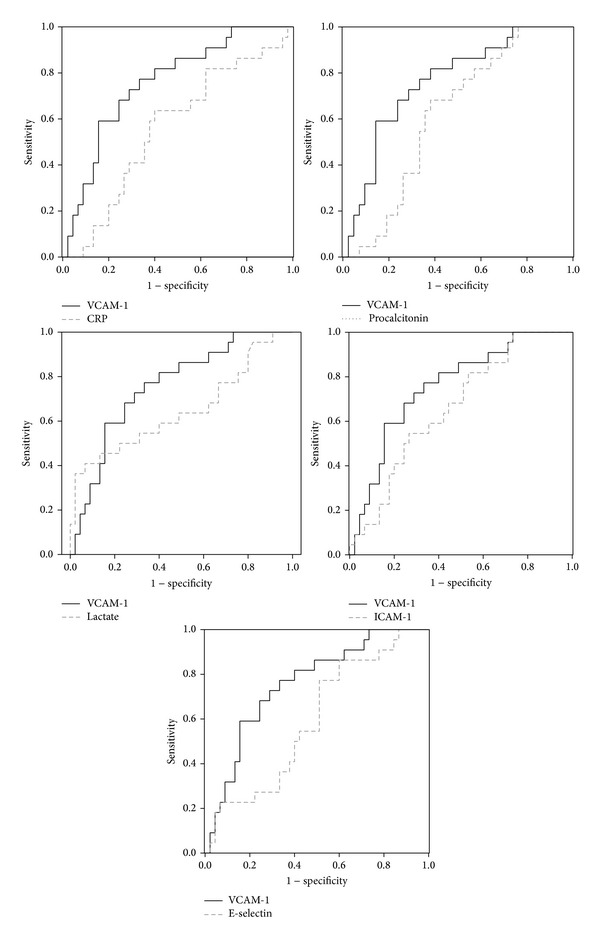
The comparison ROC curve of various biomarkers with sVCAM-1 for predicting septic encephalopathy.

The effectiveness of infection markers in predicting SE in the ER setting was evaluated by assessing the area under curve (AUC) of each biomarker's ROC curves. The AUCs for each marker were calculated (Table 4; Figure 1). The AUC for CRP, procalcitonin, lactate, E-selectin_Day1_, sICAM-1_Day1_, and sVCAM-1_Day1_ levels was 0.561 (*P* = 0.423), 0.616 (*P* = 0.130), 0.647 (*P* = 0.052), 0.593 (*P* = 0.219), 0.664 (*P* = 0.031), and 0.760 (*P* = 0.001), respectively. sVCAM-1_Day1_ level had the highest AUC, reflecting good discrimination. Our suggestion of sVCAM-1_Day1_ cut-off value for predicting SE was 1900 ng/mL for the better sensitivity (sensitivity of 81.8%, specificity of 61.9%, positive predictive value of 51.2%, and negative predictive value of 87.4%).

### 3.4. Time Course of Serum Vascular Cell Adhesion Molecule Levels

**Figure 2:**
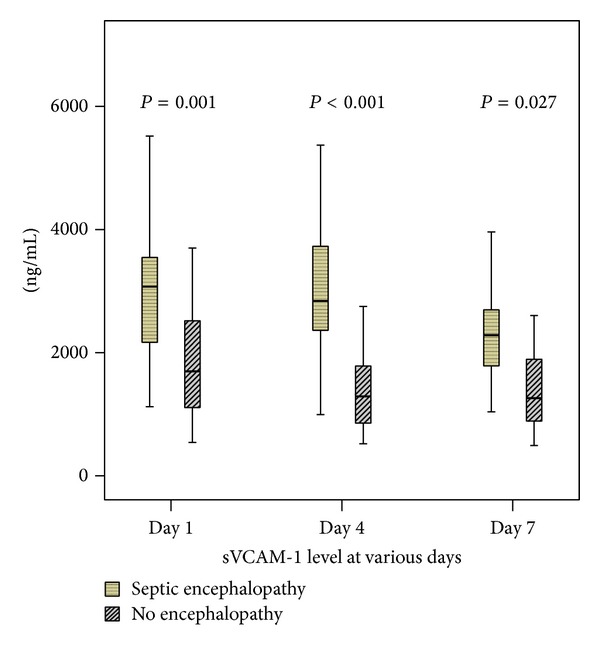
Comparison of serum VCAM-1 level between septic encephalopathy (SE) and non-SE groups in various days.

Serum sVCAM-1 levels obtained at different days (Figure 2) revealed that sVCAM-1 level gradually decreased in both groups. In all three different testing points, the SE group had significantly higher sVCAM-1 level than the non-SE group using Mann-Whitney *U* test (3048.1 ± 1261.1 versus 1969.0 ± 1129.5, *P* = 0.001; 3011.5 ± 1158.1 versus 1532.6 ± 1005.2, *P* < 0.001; 2376.4 ± 1017.9 versus 1597.6 ± 1048.0, *P* = 0.027, resp.). By repeated measures of ANOVA, serum VCAM-1 levels between the two groups at three different time points, Days 1, 4, and 7, were significantly different (*P* = 0.011).

## 4. Discussion

The present study examines the association between SE and circulatory cell adhesion molecules and produces the following major findings. First, the level of serum sVCAM-1, sICAM-1, and lactate on presentation, 24 h SOFA score, 24 h APACHE II score, underlying diseases of stroke, and ventilator treatment within 24 hours were significantly higher in the SE group of severe septic patients than in the non-SE group. Second, VCAM-1 level on presentation is a more powerful predictor of SE in severe sepsis patients than lactate concentration and other adhesion molecules on admission by stepwise logistic regression and AUC analysis. Lastly, sVCAM-1 levels in the SE group were significantly higher than those of the non-SE group at three time periods (Days 1, 4, and 7).

In this study, SE is closely related to the severity and mortality of severe sepsis and septic shock. The 24 h APACHE II score, SOFA score, serum lactate level, ventilator treatment within 24 hours, and mortality rate in SE of severe septic patients are significantly higher than those of non-SE patients. The longer duration of SE also implies higher mortality since four patients died within three more days of SE. Our result is compatible with other studies [30]. In another study, they also found that mortality related to SE increased from 16% to 63% when the GCS decreased from 15 to less than 8 [12]. The increased mortality in SE group could partially explained by increased severity of sepsis. But further evaluation of the direct effect of brain injury to the systemic response may need to clarify it.

Patients with SE had also higher incidence of early respiratory failure. In clinical practice, patients with conscious disturbance are at risk of respiratory failure since they have less airway protection and respiratory drives. Certain periods of hypoxemia before respiratory failure may also cause more brain injury. Tissue hypoperfusion can lead to elevated serum lactate level and increased acidosis, which can explain part of the mechanisms of SE by their correlation.

Cell adhesion molecules regulate endothelial function by activating leukocyte recruitment and tissue inflammation. Endothelium dysfunction may be central to the development of sepsis-induced multiple organ failure [31]. Soluble form of VCAM-1, ICAM-1, and E-selectin are present in plasma and reflect cellular inflammatory status and correlate with endothelial dysfunction [32]. In the present study, all three kinds of cell adhesion molecules and serum lactate correlate with SOFA score. They can represent the degree of organ damage in severe sepsis and septic shock patients.

Brain has a unique blood-brain barrier that regulates the brain capillary blood flow and thus precisely maintains the brain internal microenvironment [33]. But endothelial activation may result in the breakdown of the blood-brain barrier [34]. Increased permeability of the blood-brain barrier has been demonstrated in experimental models of sepsis [35]. The brain MRI of SE patients also reveals vasogenic edema of the brain parenchyma [36, 37]. Hofer's study using septic animal models reveals early changes in the integrity of the blood-brain barrier in the central nervous system. Increased cerebral ICAM-1 expression may be an early factor involved in these pathogenic events [20]. In Hamed's study, children with sepsis-induced encephalopathy have elevated serum and cerebrospinal fluid levels of sICAM-1, NO, and S100B compared to those with sepsis only [38]. Both studies used only sICAM-1 as the biomarkers.

In our study, both sICAM-1 and sVCAM-1 had correlated with SE. Serum sVCAM-1 and sICAM-1 are also correlated with each other in this study. Previous study has revealed that many signaling events attributed to ICAM-1 engagement appear to be similar to VCAM-1 [39]. It is difficult to disentangle the two CAMs effects. In cardiovascular disease study, sICAM appears to be a general marker of proinflammation and may be used as a risk factor. But sVCAM-1, which is not expressed in baseline conditions, is rapidly induced by injury and can emerge as a strong risk predictor of existing disease [40]. Compared to sICAM-1 and E-selectin, it is sVCAM-1 that has the strongest correlation with cardiovascular-related future death and cardiovascular events [41]. Just like our study, sVCAM-1 is also a better predictor than other adhesion molecules in SE patients.

### 4.1. Limitations

Although this study demonstrates that serum VCAM-1 level on presentation is a more powerful predictor of SE in severe sepsis patients than lactate concentration and other adhesion molecules on admission, this study has several limitations. First, the occurrence of SE can be at the time of presentation or after the stay at the ER and both the duration and severity of SE were different in each patient. Its relationship with levels of serum cell adhesion molecules cannot be exactly evaluated in every SE patient. Second, although only nontraumatic, nonsurgical septic patients are enrolled and patients with underlying brain pathologies, cognitive decline, and continuous sedative medication use have been excluded, not all of the severe sepsis patients received both neuroimaging studies and electroencephalography studies. The findings may underestimate the “true” frequency of SE in septic patients in this study. Lastly, the choice of therapeutic strategy for sepsis (e.g., use of steroids and choice, dosage, and duration of antibiotics) may be different for each patient based on the preference of the attending physician. This may cause potential bias in patients' outcome and serum biomarkers levels.

## 5. Conclusion

In conclusion, this study demonstrates that SE implies higher mortality in severe septic patients and VCAM-1 level on presentation is a more powerful predictor of SE than lactate concentration and other adhesion molecules on admission.

## Figures and Tables

**Table 1:** Baseline characteristics of septic encephalopathy (SE) and non-SE groups in severe sepsis patients.

	SE group *n* = 23	Non-SE group *n* = 47	*P* value
Age (y) (mean ± SD)	68.0 ± 11.2	62.6 ± 13.6	0.15
Male/female	17/6	31/16	0.59
Underlying diseases [*n* (%)]			
Diabetes mellitus	10 (44)	15 (32)	0.43
Hypertension	8 (35)	22 (47)	0.44
Liver diseases/alcoholism	6 (26)	6 (13)	0.19
Chronic lung disease	3 (13)	11 (23)	0.36
Stroke^a^	7 (30)	4 (9)	0.03*
Coronary artery disease	0 (0)	6 (13)	0.17
Cancer	6 (21)	10 (21)	0.76
Chronic renal disease	2 (9)	1 (2)	0.25
Clinical presentations (mean ± SD)			
Systolic BP	108.0 ± 39.0	108.0 ± 47.0	0.91
Diastolic BP	72.1 ± 24.2	64.2 ± 22.8	0.20
Pulse rate	103 ± 26	112 ± 25	0.26
Respiratory rate	20 ± 3	21 ± 4	0.25
Shock within 24 hours [*n* (%)]	15 (65)	34 (74)	0.58
Ventilator treatment within 24 hours [*n* (%)]	13 (57)	11 (24)	0.008*
Disease severity index (mean ± SD)			
Maximum 24 h APACHE II score	21.3 ± 5.5	17.5 ± 5.7	0.01*
CCI score	3.6 ± 2.6	3.2 ± 3.0	0.44
Maximum 24 h SOFA score	8.2 ± 2.4	5.4 ± 3.1	<0.001*
Steroid treatment [*n* (%)]	11 (48)	21 (45)	1.00
In-hospital mortality [*n* (%)]	9 (40)	5 (11)	0.009*

SD: standard deviation; BP: blood pressure; APACHE: Acute Physiology and Chronic Health Evaluation; CCI: Charlson Comorbidity Index; SOFA: Sequential Organ Failure Assessment.

^a^Stroke only referred to the patients who still could maintain clear conscious level after ischemic or hemorrhagic stroke event.

*P < 0.05.

**Table 2:** Sources of infection in septic encephalopathy (SE) and non-SE groups.

Sources of infection	SE group *n* = 23	Non-SE group *n* = 47	*P* value
Respiratory tract infection	8 (36.4)	19 (40.4)	0.955
Urinary tract infection	4 (18.2)	9 (19.1)	
Intra-abdominal infection	4 (18.2)	10 (21.3)	
Soft tissue infection	5 (22.7)	7 (14.9)	
Unknown origin	1 (4.5)	2 (4.3)	

Concurrent bacteremia episode	13 (56.5)	21 (44.7)	0.192

Causative pathogens			0.56
Gram-negative			
*Escherichia coli *	5 (21.7)	6 (12.8)	
*Klebsiellapneumoniae *	2 (8.7)	8 (17.0)	
*Proteus mirabilis *	1 (4.3)	1 (2.1)	
*Burkholderiapseudomallei *	1 (4.3)	0 (0)	
*Salmonella enteritidis *	1 (4.3)	0 (0)	
Gram-positive			
*Streptococcus pneumoniae *	0 (0)	2 (4.3)	
**β*-Hemolytic Streptococcus group A *	1 (4.3)	2 (4.3)	
*Staphylococcus aureus *	2 (8.7)	2 (4.3)	

**Table 3:** Laboratory data of the septic encephalopathy (SE) and non-SE groups in severe sepsis patients.

	SE group *n* = 23	Non-SE group *n* = 47	*P* value
WBC/DC (mean ± SD)			
WBC (×10^9^/L)	15.0 ± 10.0	17.7 ± 12.0	0.13
Segment (%)	76.7 ± 20.3	81.8 ± 17.3	0.38
Band (%)	8.5 ± 12.7	3.2 ± 4.8	0.06
Lymphocyte (%)	7.7 ± 7.9	10.4 ± 17.4	0.96
Biochemistry (mean ± SD)			
Glucose (mg/dL)	210 ± 162	171 ± 94	0.91
Creatinine (mg/dL)	2.9 ± 1.9	2.3 ± 2.5	0.06
Lactate (mg/dL)	50.5 ± 37.6	32.6 ± 20.3	0.05*
Total bilirubin (mg/dL)	3.3 ± 3.0	2.2 ± 2.7	0.09
BNP (pg/mL)	991 ± 1127	742 ± 1024	0.21
Inflammatory markers (mean ± SD)			
CRP (mg/L)	231 ± 112	206 ± 137	0.40
Procalcitonin (ng/mL)	32.5 ± 33.4	35.6 ± 59.4	0.12
sICAM-1_Day1_ (ng/mL)	1028.2 ± 525.2	764.8 ± 504.9	0.03*
sVCAM-1_Day1_ (ng/mL)	3048.1 ± 1261.1	1969.0 ± 1129.5	0.001*
sP-selectin_Day1_ (ng/mL)	98.8 ± 42.5	98.0 ± 23.0	0.77
sL-selectin_Day1_ (ng/mL)	1208.2 ± 777.5	933.9 ± 311.9	0.10
sE-selectin_Day1_ (ng/mL)	214.2 ± 111.9	117.8 ± 127.6	0.22

WBC: white blood cells; DC: differential count; CRP: C-reactive protein; BNP: B-type natriuretic peptide; sE-selectin: soluble E-selectin; sICAM-1: soluble intercellular adhesion molecule-1; sL-selectin: soluble L-selectin; sP-selectin: soluble P-selectin; sVCAM-1: soluble vascular cell adhesion molecule-1.

*P < 0.05.

**Table 4:** AUC for biomarkers in diagnosing septic encephalopathy by ROC curve analysis.

	AUC	95% confidence interval	*P* value
CRP	0.561	0.416–0.705	0.423
Procalcitonin	0.616	0.479–0.752	0.130
Lactate	0.647	0.494–0.800	0.052
sVCAM-1	0.760	0.638–0.881	0.001*
sICAM-1	0.664	0.532–0.795	0.031*
sE-selectin	0.593	0.452–0.734	0.219

AUC: area under the curve; CRP: C-reactive protein; sVCAM-1: soluble vascular cell adhesion molecule-1; sE-selectin: soluble E-selectin; sICAM-1: soluble intercellular adhesion molecule-1; ROC: receiver operating characteristic.

*P < 0.05.
